# Sex-Specific Crossover Distributions and Variations in Interference Level along Arabidopsis thaliana Chromosome 4

**DOI:** 10.1371/journal.pgen.0030106

**Published:** 2007-06-29

**Authors:** Jan Drouaud, Raphaël Mercier, Liudmila Chelysheva, Aurélie Bérard, Matthieu Falque, Olivier Martin, Vanessa Zanni, Dominique Brunel, Christine Mézard

**Affiliations:** 1 Station de Génétique et d'Amélioration des Plantes, Institut Jean Pierre Bourgin, INRA, Versailles, France; 2 UR Etude du Polymorphisme des Génomes Végétaux, Centre National de Génotypage, Evry, France; 3 UMR de Génétique Végétale, INRA, Université Paris-Sud, CNRS, Institut National Agronomique Paris-Grignon, Gif-sur-Yvette, France; 4 Laboratoire de Physique Théorique et Modèles Statistiques, Université Paris-Sud, Orsay, France; National Cancer Institute, United States of America

## Abstract

In many species, sex-related differences in crossover (CO) rates have been described at chromosomal and regional levels. In this study, we determined the CO distribution along the entire Arabidopsis thaliana Chromosome 4 (18 Mb) in male and female meiosis, using high density genetic maps built on large backcross populations (44 markers, >1,300 plants). We observed dramatic differences between male and female map lengths that were calculated as 88 cM and 52 cM, respectively. This difference is remarkably parallel to that between the total synaptonemal complex lengths measured in male and female meiocytes by immunolabeling of ZYP1 (a component of the synaptonemal complex). Moreover, CO landscapes were clearly different: in particular, at both ends of the map, male CO rates were higher (up to 4-fold the mean value), whereas female CO rates were equal or even below the chromosomal average. This unique material gave us the opportunity to perform a detailed analysis of CO interference on Chromosome 4 in male and female meiosis. The number of COs per chromosome and the distances between them clearly departs from randomness. Strikingly, the interference level (measured by coincidence) varied significantly along the chromosome in male meiosis and was correlated to the physical distance between COs. The significance of this finding on the relevance of current CO interference models is discussed.

## Author Summary

Meiotic crossovers between homologous chromosomes ensure their proper segregation to generate ultimately gametes. They also create new allelic combinations which contribute to the diversity of traits among individuals. In all eukaryotes, the number and the localization of crossovers along chromosomes are not random. In addition, crossovers are not independent of each other: the occurrence of a crossover lowers the probability that another crossover arises in its vicinity. The mechanism of this phenomenon, called “crossover interference,” is one of the most challenging puzzles that geneticists have been faced with in the last century. In this paper, we precisely described the distribution of crossovers along Chromosome 4 of the model plant species *Arabidopsis thaliana,* separately in male and female meiosis. Interestingly, we observed that crossovers are 1.7 more numerous in male than in female meiosis, and this increase is especially marked at the ends of the chromosome. Moreover, our results provide the first evidence that the level of interference along a chromosome is not a constant and is correlated with the physical distance between crossovers. These results shed new light on the determinism of crossover localization and could have important outcomes on the relevance of current models of crossover interference.

## Introduction

One prominent feature of the eukaryotic life cycle is the segregation of homologous chromosomes to two different cells during the first, also known as reductional, meiotic division. The proper completion of this segregation relies on the formation of stable physical connections between homologous chromosomes. In most eukaryotic species, these connections are mediated by crossovers (COs). These are sites where large (megabase scale) segments of homologous (nonsister) chromatids are exchanged. Consequently, COs are essential to the ploidy reduction process, as well as to play a role in the creation of allelic combinations.

CO number and distribution along chromosomes differ between male and female meiosis in many plant and animal taxa (for review see [1]). This widespread phenomenon is called heterochiasmy. Both the direction and magnitude of these differences are highly variable. For example, depending on the species, CO number may be higher in female (F) meiosis (most eutherian mammals), or male (M) meiosis (some metatherian mammals), or there may be no significant difference between sexes (goat, dog, barley). This difference may be small or moderate, but sometimes it is huge (e.g., teleostean fishes). Even closely related species can exhibit different M/F CO ratios. In the *Brassicaceae,* for example, this ratio reaches 1.2 in Sinapis alba [2], whereas in Brassica oleracea it is inversed (0.6) [3], and there is no significant difference in Brassica napus (0.98) [4]. Therefore, the nature of evolutionary forces driving heterochiasmy is a puzzling issue. In addition, the underlying molecular and cellular mechanisms are currently unknown. Yet sex-related differences in CO number per chromosome are paralleled by sex-related differences in the length of synaptonemal complex (SC) in human [5] and mouse [6]. The SC is a proteic structure scaffolded along synapsed homologous chromosomes at pachytene stage [7].

COs can be localized along chromosomes by analyzing genetic recombination data. They can also be visualized cytologically either as chiasma, or as immunolabeled MLH1 foci that mark most CO sites [8], or as late recombination nodules [9], which are electron-dense structures located on SCs [9–11]. COs originate from programmed double-strand breaks (DSBs) that occur early in prophase of the first meiotic division [12]. Only a part of these DSBs give rise to COs; the remaining DSBs are repaired as “noncrossovers” (NCOs), without exchange of large DNA segments between homologous chromosomes.

Numerous studies showed that CO formation is tightly controlled at both chromosomal and local scales [13,14]. Indeed, COs are not uniformly distributed and inter-CO distances are not random. The former feature is well illustrated by numerous datasets in mammals [15–17] and higher plants [11,18]. Several studies have tried to correlate CO rates along chromosomes with various sequence features, such as gene or transposable element density, GC nucleotides %, CpG ratio, simple repeats, etc. However, even if some weak correlations were found, it seems that none holds true in all species [15,16,19–21], suggesting that other constraints act on CO distribution.

One of these constraints is CO interference. This phenomenon was originally described as a lower frequency of double-COs in disjoint chromosomal segments than expected if they occur independently of each other [22]. The existence of interference has been confirmed in most species tested [10]. As a consequence of interference, COs tend to be more evenly spaced than expected if CO positions were random [23]. In addition, in many species, which have a limited number of COs per chromosome, interference tends to increase physical distances between adjacent COs. This is well illustrated by recombination nodules or MLH1 foci maps produced in various species [5,17,24–26].

The mechanisms of interference setup are still poorly understood. Several models of meiotic CO interference have been proposed over years (see [14] for a comprehensive review). The two main contenders are currently the “counting” model [27] and the mechanical stress model [28]. The basic postulate of the counting model is that the CO designation process among recombination precursors occurs in such a way that any two adjacent COs are separated by a fixed number of NCOs. Alternatively, the mechanical stress model hypothesizes that COs originate from a mechanical stress imposed on the chromosome. CO designation would promote a stress relief that would (i) inhibit CO designation among nearby recombination intermediates and (ii) attenuate in a distance-dependent manner. Neither of these two models is presently strongly supported by experimental data.

In a previous study, we produced a high resolution map (at around the 210-kb scale) of meiotic crossovers on Arabidopsis thaliana Chromosome 4 [18]. We showed that CO rates vary greatly along the chromosome from 0 to 20 cM/Mb, and that COs displayed interference. However, CO rates on this map were sex-averaged because we used the selfed progeny of F1 hybrids for the mapping population. Given that the existence of heterochiasmy in A. thaliana had been previously suggested by several studies [29–32], we decided to investigate the relative contributions of male and female meiosis in the distribution of COs on Chromosome 4. We observed dramatic differences between male and female genetic maps. Strikingly, we found a good correlation between the sex-ratio of mean CO number per Chromosome 4 on one hand and the sex-ratio of total SC length on the other hand. Moreover, we were able to detect significant variations in interference strength along Chromosome 4. Stunningly, it turned out that interference strength covaries with the physical distance between COs. These results could have important upshots on the reliability of current interference models.

## Materials and Methods

### Generation of Backcross Populations and Genomic DNA Extraction

A. thaliana “Columbia” (Col) and “Landsberg erecta” (Ler) accessions were crossed to obtain F1 hybrids. Col plants were then crossed with an F1 hybrid used either as the male (Col × (Col × Ler)) or as the female ((Col × Ler) × Col) parent. Seeds from these crosses were sowed in vitro, and then seedlings were grown in short-day conditions at 21 °C.

After 2 wk, 1,476 whole seedlings of each population were collected and their DNA was extracted as described previously [33].

### Choice of Markers and Single Nucleotide Polymorphism Genotyping

In a previous experiment, F2 plants from a Col × Ler cross were genotyped with a set of 70 SNP markers spanning A. thaliana Chromosome 4 [18]. In the present study, 46 SNPs out of these 70 and two additional SNPs were chosen so that the mean sex-averaged genetic distance between adjacent markers was 1.9 cM. SNPs are listed in Table S1.

Genotyping was performed using SNPlex technology (Applied Biosystems, http://www.appliedbiosystems.com) following the supplier protocols. After quality scoring of genotyping data, four markers were dismissed from the whole dataset. In some cases it was not possible to assess the genotype of remaining markers in some plants so these were also removed from the dataset. The resulting populations comprised 1,305 and 1,419 plants for female and male meiosis, respectively.

The genetic size of intervals was computed as the ratio between the number of recombined chromosomes and the number of analyzed meioses, which in the case of a backcross progeny is equal to the number of analyzed plants. CO rates, physical and genetic sizes are listed in Table S2.

### Comparisons of CO Rates

In order to calculate single-interval, sex-averaged CO rates in the pool of M and F populations, the sex-specific CO rates were weighted according to the respective population size.

All pair-wise comparisons between CO rates were performed using a chi-square homogeneity test. For multiple testing, *p*-values were subsequently corrected using the false discovery rate procedure [34].

### Comparisons of CO Number per Chromosome

Predicted Poisson distributions of CO number per chromosome were calculated using the following formula: 
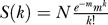
 where *S*(*k*) is the number of chromosomes harboring exactly *k* CO, *e* is the neperian logarithm base, *m* is the observed mean number of CO per chromosome, and *N* is the total number of chromosomes.

Whole comparisons between observed and Poisson distributions were performed using a chi-square goodness-of-fit test.

### Comparisons of Inter-CO Distances

For both M and F datasets, the genetic “width” of inter-CO distance classes was chosen to be 17.5 cM (± 5%) in order to: (i) provide distance classes spanning the whole chromosome genetic length, (ii) ensure a common denominator in both M and F maps, and (iii) prevent small class sizes, in order to maintain moderate sampling variances, thus allowing conclusive statistical testing.

The continuous probability distribution function of inter-CO distances on chromosomes with exactly *a* independent COs is: 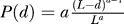 *,* where *L* is the genetic size of the chromosome and *d* is the distance between successive COs. The derivation of this formula is as follows:

*a* independent CO points are randomly placed on a chromosome of length *L.* Then a CO point is added at one end to bring the chromosome to the shape of a ring. *a* + 1 points are thus randomly and independently positioned on the circle of perimeter *L.* The statistics of distances between successive COs is the same for all pairs; to compute this for the first pair, we need to find the distribution of the smallest of *a* random variables, representing the positions of COs along the interval, which are uniformly distributed in [0, *L*] (the remaining point is by definition at position zero). The probability that this smallest value, which is the distance between the last and the first CO, is greater or equal to *X* is (1 − *X*/*L*)*^a^.* The minus derivative of this cumulated distribution then gives the desired probability distribution.

The following formula, which is easily deduced from the formula above, allows convenient calculation of discrete distributions of inter-CO distances on finite-size chromosomes with exactly two independent COs: 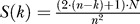 *,* where *n* is the number of classes, *k* is the rank of the class (increasing with distance), *N* is the population size, and *S*(*k*) is the size of the k^th′^ class.

Whole comparisons between observed and calculated distributions were performed using a chi-square goodness-of-fit test.

### Coincidence Analyses

For both M and F datasets, the genetic size of intervals used for coincidence analyses was chosen to be the same as the genetic “width” of distance classes used for inter-CO distance comparisons, for the same reasons (see above). Given two intervals, the coefficient of coincidence between them is calculated as follows: 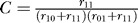 where *C* is the coincidence and *r*_ij_ is the chance of i CO across the first interval and j CO across the second interval. In most cases i and j values are either 0 or 1; 2 COs were rarely found in one of the intervals and were considered as no CO, while 3 COs were considered as 1 only, accordingly to what would have been observed if the intervals would have not contained internal markers.

The standard deviation of coincidence was calculated according to [35].

### Testing for Significant Differences between Coincidence Values

We have developed a procedure which computes the *p*-value for the hypothesis H_0_ that two coefficients of coincidence *c_u_* and *c_v_* estimated from quadruplets or triplets of markers are in fact generated from the same theoretical coincidence value *c_th_.* Under that hypothesis, H_0_, *c_u_,* and *c_v_* are actually not expected to differ from each other. A small *p*-value for the difference between *c_u_* and *c_v_* then indicates that H_0_ is unlikely to be true given the genotype data of the mapping population.

Let a quadruplet have markers A, B, C, and D, assumed to be in the order in which they appear on the chromosome. If the quadruplet is instead a triplet, this formalism can be applied by setting B = C.

In a first phase, we compute *c_th_* by the maximum likelihood method. Consider the first quadruplet: the probability (likelihood) that *N* gametes lead to a measured coincidence value of *c_u_* is

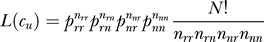
where *n_nn_, n_rn_, n_nr_, n_rr_* are the number of gametes that are respectively recombinant between (i) neither A and B nor C and D, (ii) A and B but not C and D, (iii) C and D but not A and B, (iv) both A and B and C and D. *N* is the total number of gametes with valid data at the four markers, namely *n_nn_*+ *n_rn_*+ *n_nr_*+ *n_rr._* The dependence on *c_th_* is through the probabilities:

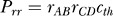


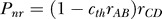


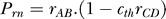


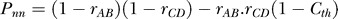
where *r_AB_* and *r_CD_* are recombination fractions between A and B and B and C, respectively.

Next, we consider the two quadruplets of interest. *L*(*c_v_*) is calculated as for *L*(*c_u_*). The joint likelihood of both observations is the product *L*(*c_u_*) × *L*(*c_v_*), and we numerically determine the *c_th_* which maximizes this joint likelihood. The result is a *c_th_* lying somewhere between *c_u_* and *c_v_.*

In a second phase, we compute a *p*-value for the hypothesis H_0_ given *c_th_.* We do this by determining the probability that | *c_u_* − *c_v_* | is at least as large as measured from the experimental data. But if *c_u_* and *c_v_* are estimated using shared gametes, the recombination events in the four intervals are a priori correlated. Thus, when measuring *c_u_* and *c_v_* we need to use independent sets of gametes by using half (*N*/2) of the gametes for *c_u_* and the other half for *c_v_.* So that the value | *c_u_* − *c_v_* | is not dependent on the data order, | *c_u_* − *c_v_* | is computed for 10^5^ random order combinations and the median value taken.

The *p*-value is obtained by simulating interference events within H_0_ given *c_th_:* we generate *N*/2 realizations of gametes for each quadruplet; for each realization, we choose among the four possibilities of recombinants or not in each interval according to the probabilities *p_rr_, p_rn_, p_nr_, p_nn_.* For this set of *N* gametes, we extract the two associated coincidence coefficients *c_u_′* and *c_v_′.* Repeating this 10^5^ times, we get a probability distribution for | *c_u_′* − *c_v_′* |; the desired *p*-value is then the frequency with which | *c_u_′* − *c_v_′* | is larger than the experimental value.

### Cytological Observations

Cytological observations were carried out on Col × Ler F1 plants.

The anti-ASY1 polyclonal antibody has been described elsewhere [36]. It was used at a dilution of 1:500. The anti-ZYP1 polyclonal antibody was described by [37]. It was used at a dilution of 1:500.

Preparation of prophase stage spreads for immunocytology was performed according to [36] with the modifications described in [38].

All observations were made using a Leica (http://www.leica.com) DM RXA2 microscope; photographs were taken using a CoolSNAP HQ (Roper, http://www.roperscientific.com) camera driven by Open LAB 4.0.4 software; all images were further processed with Open LAB 4.0.4 or AdobePhotoshop 7.0 (http://www.adobe.com). SC length measurement was performed using Optimas (Bioscan Incorporated, http://www.bioscan.com) software.

## Results

Plants from a Col × Ler F1 population were backcrossed with Col plants using the F1, either as the male or the female parent, in order to create two populations subsequently referred to as M and F, in which the observed recombination events occurred either in male or female meiosis of the parental F1 hybrid. 1,419 M plants and 1,305 F plants were genotyped with 44 SNP markers spanning Chromosome 4 at a density of 1.9 cM (calculated from sex-averaged data, see Materials and Methods). Given that interval sizes are small, we calculated genetic distances simply by dividing the number of recombinant chromosomes by the total number of plants analyzed.

### The CO Landscape on Chromosome 4 Does Not Differ between an F2 Population and Pooled Backcross Populations

**Figure 1:**
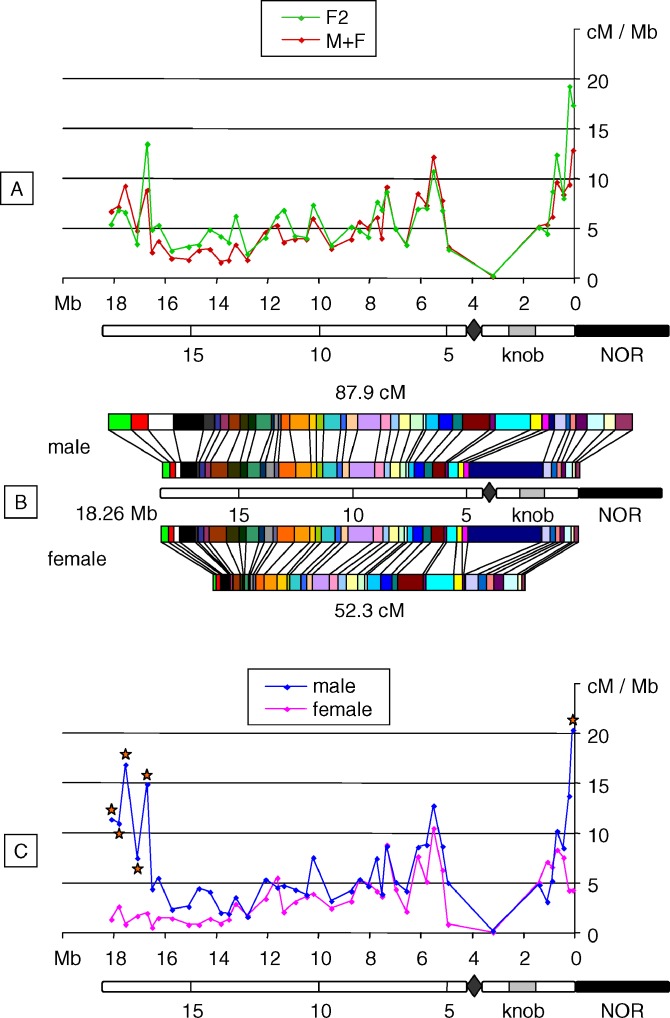
Variation of CO Rates along A. thaliana Chromosome 4 (A) CO rates in F2 population (green) and the pool of male and female backcross populations (red). (B) Alignment of physical map (center) and both male (top) and female (bottom) genetic maps. (C) CO rates in male (blue) and female (pink) populations. Orange stars mark the intervals that are significantly different between both populations (*p*-values < 0.02). A schematic representation of Chromosome 4 is aligned with the physical map and each CO plot, which includes 5-Mb scale coordinates, centromere (diamond), heterochromatic knob (gray box), nucleolar organizer region (NOR, black box).

We first compared CO rates in the F2 population previously described to those in pooled M and F populations, in each of the same 43 intervals spanning Chromosome 4 (Figure 1A). As expected, the “averaged” (see Materials and Methods) CO rates observed in the pool of the M and F backcross progenies (corresponding respectively to male and female meiosis) were not significantly different from those observed in the F2 progeny (resulting half from male meiosis, half from female meiosis) generated from the same parental accessions (lowest *p*-value is 0.35; Figure 1A).

This implies that there is no significant variation in meiotic recombination over time for a given genetic background, thus enabling direct comparisons of data.

### The CO Landscape on Chromosome 4 Differs in Male and Female Meiosis

At first glance, the difference between male and female recombination rates is obvious when comparing total genetic size of both maps (Figure 1B). The M map is 87.9 cM long and the F map is 52.3 cM long. This indicates that a Chromosome 4 bivalent experiences on average 1.76 CO in male meiosis, but only 1.05 CO in female meiosis (M/F ratio 1.68). This M/F difference is highly significant (χ^2^ *p*-value < 0.001)

Next, we compared recombination rates in male and female meiosis interval-by-interval (Figure 1C). For a majority of intervals (36/43) the M/F ratio was above 1, with the most notable differences in the last telomeric third of the long arm. However, only the distal interval on the short arm and the five distal intervals on the long arm were highly significantly different in male and female (mean M/F ratio for these six intervals is 6.1, χ^2^ *p*-value < 0.05). The remaining central intervals were not significantly different in male and female, when compared one-by-one. However, if these were grouped and considered as a single interval, there was still a significant difference between male and female (M/F ratio 1.37, χ^2^ *p*-value < 0.001).

In summary, male and female meiotic CO landscapes along Chromosome 4 are strikingly different. The difference is high close to the telomere on the long arm and to the nucleolar organizer region on the short arm and modest in the median region of the chromosome (see Figure 1B and 1C).

### Total SC Length Differs in Male and Female Meiosis

**Figure 2:**
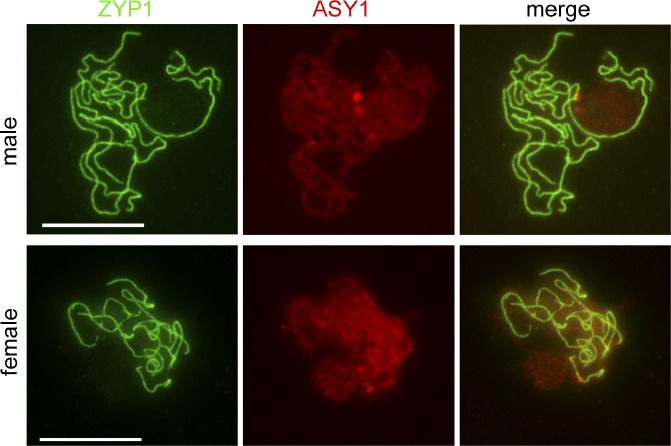
Coimmunolocalization of ASY1 (Red) and ZYP1 (Green) in Male and Female Meiocytes at Pachytene Stage Bar = 10 μm.

Meiotic chromosomes at pachytene stage were immunolabeled with antibodies against ZYP1. This protein is a major component of the central element of the SC, which ties homologous chromosomes together. We used ASY1 immunolabeling to visualize the axial element, which is a proteinaceous axis formed along pairs of sister chromatids [39] (Figure 2). At pachytene stage, ZYP1 labeling extends continuously along the entire SC, hence allowing total SC length measurement. We found that SC length in male meiocytes is 166 ± 24 μm (*n* = 22) compared to only 98 ± 20 μm (*n* = 25) in female. Our estimate of male SC length is in good agreement with that obtained in a previous study (147 ± 28 μm; *n* = 19) using electron microscopy [40]. The value we obtained for the M/F ratio of total SC lengths is very close to that for the M/F ratio of mean CO numbers per chromosome (1.70 versus 1.76). This suggests that sex-related differences in CO number and total SC length are correlated.

### Distributions of CO Number per Chromosome in Male and Female Meiosis Do Not Fit Poisson Distributions.

**Figure 3:**
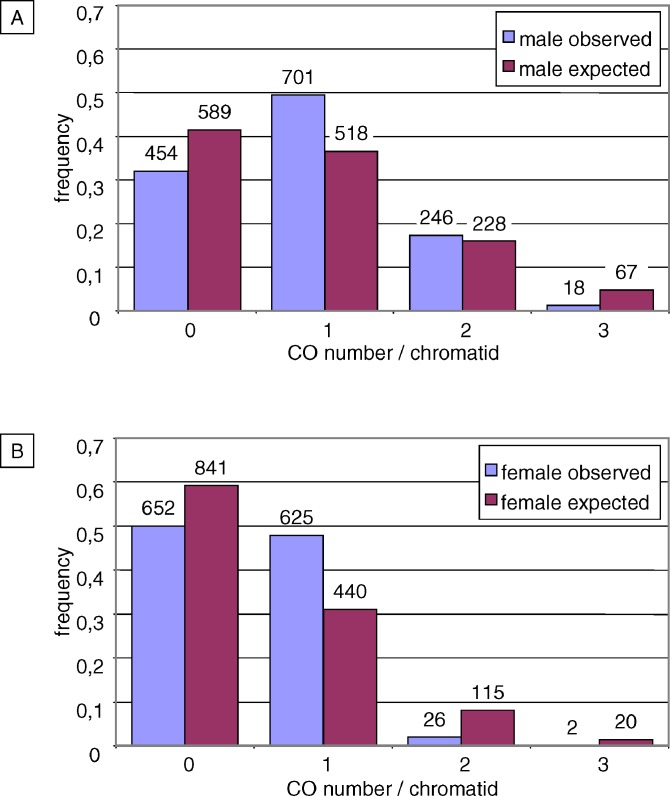
Distributions of CO Number per Chromosome 4 Bars represent the frequency of chromatids with 0, 1, 2, or 3 COs. (A) Observed (blue) and Poisson (purple) distributions in M population. (B) Observed (blue) and Poisson (purple) distributions in F population. Corresponding number of chromatids is indicated above each bar.

We next looked at the distribution of CO number per Chromosome 4 in the M and F populations (Figure 3). According to the hypothesis that CO placements are random and independent events, the distribution of CO number per chromosome should fit a Poisson distribution. Thus, we calculated the Poisson distributions expected for the observed average number of COs per Chromosome 4 and compared these to the observed ones (see Figure 3A and 3B).

In the F population, about half of chromosomes had no CO or only one CO, while very few had two COs or more (Figure 3B). This distribution is highly significantly different (*p*-value < 0.001) from the theoretical Poisson distribution, in which the “0 CO” group was the main class (59%) and multiple CO classes accounted for 10%. Hence, in female meiosis almost all bivalents experienced only the “obligate CO” required for the proper segregation of homologous chromosomes at anaphase I.

In the M population, only one third of chromosomes had no CO and about half had one CO (Figure 3A). Consequently, chromosomes with multiple COs were more frequent than in the F population. Conversely, in the corresponding Poisson distribution “0 CO” and “1 CO” chromosomes were represented at 42% and 36%, respectively. Observed and expected distributions were clearly different from each other (*p*-value < 0.001).

### Distances between Adjacent COs Are Greater than Expected

**Figure 4:**
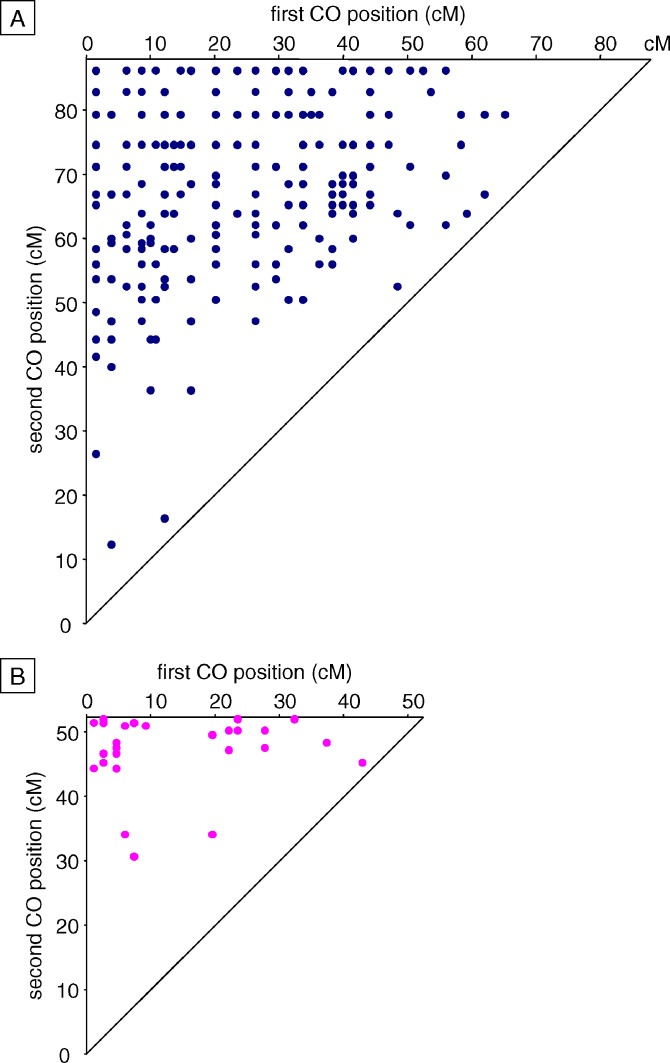
Double-CO Locations on (A) Male and (B) Female Chromosome 4 All double-COs on chromosomes harboring exactly two COs were plotted in two-dimensional graphs. The x and y axis values indicate the genetic position (relative to the nucleolar organizing region) of the first and the second CO, respectively. The diagonal line and the upper left corner correspond to minimal (null) and maximal (chromosome-wide) inter-COs distances, respectively.

As a consequence of interference, inter-CO distances are less variable and greater (when CO number is limited) than expected under the assumption that COs are distributed randomly and independently. Positions of double-COs (on chromosomes with exactly two COs) were represented in two-dimensional plots in Figure 4. x and y axis coordinates correspond to the positions of the first and second CO on the genetic map, respectively. Under the assumption of no interference, points should be uniformly distributed over the triangle. For both M and F double-CO populations, the observed points were clearly heterogeneously distributed: they were underrepresented next to the diagonal line, which corresponds to low inter-CO distances.

**Figure 5:**
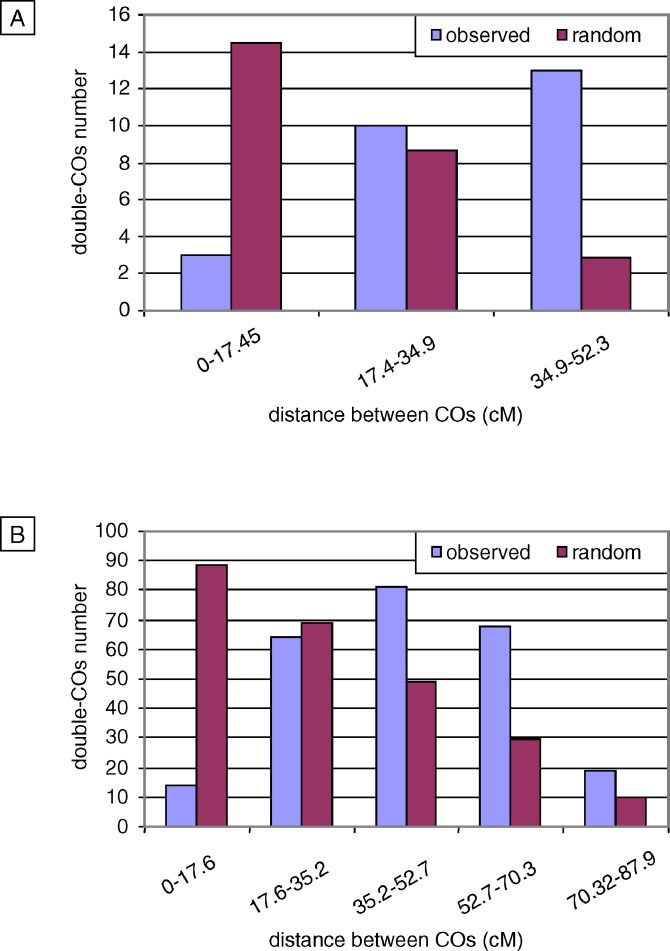
Distributions of Inter-CO Distances on Chromosome 4 Observed (blue) and random (purple) distributions of inter-CO distances in (A) F and (B) M populations.

In order to test this deviation from independence between COs, inter-CO distances on chromosomes with two COs only (see Materials and Methods) were grouped into size classes, and the observed distributions were compared to the “random” (no CO interference) distributions (Figure 5). For both M and F datasets, the genetic length (17.5 cM ± 5%) of the intervals was chosen to optimize the number of double-COs per interval, in order to avoid high sampling variance and thus allow statistically significant differences to be detected.

In the F population, we found opposing observed and expected distributions: for the expected distribution the minor class was 35–52.5 cM and the major class 0–17.5 cM, whereas in the observed distribution the majority of inter-CO distances were long, and short distances were the minority (Figure 5A). This difference was highly significant (*p*-value < 0.001).

The observed M distribution was rather symmetrical, with the mode between 35 and 53 cM. It was strikingly different from the theoretical distribution, in which the class size decreased with increasing genetic length (*p*-value < 0.001; Figure 5B).

For both M and F distributions the mean observed inter-CO distance, respectively 51% and 63% of the total map size, exceeded the expected one, which is exactly one third of the total map size.

Therefore, in male and female meiosis, widely spaced COs were overrepresented, whereas closely spaced COs were underrepresented. This difference between expected and observed distributions of distances between COs is fully consistent with interference.

### Three-Point Coefficient of Coincidence Varies along Chromosome 4

Besides altered inter-CO distances, another expected consequence of interference is a lowered chance of finding close double-COs than expected from randomness. More precisely speaking, given two intervals, double-COs (one CO in each interval) will occur at a lower frequency than two independent COs (one CO in the first or in the second interval, both being not exclusive). This departure is called coincidence and can be calculated as follows: 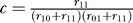 where *r*_ij_ is the chance of i CO across the first interval and j CO across the second interval. The value of C is 1 if there is no interference and 0 if interference is absolute (meaning that double-COs are completely absent). Coincidence is widely used as a measure of interference from genetic data. Moreover, most mathematical models of CO interference assume a covariation between coincidence at a given genetic distance and the level of interference (see for example [27], reviewed in [14] ).

**Figure 6:**
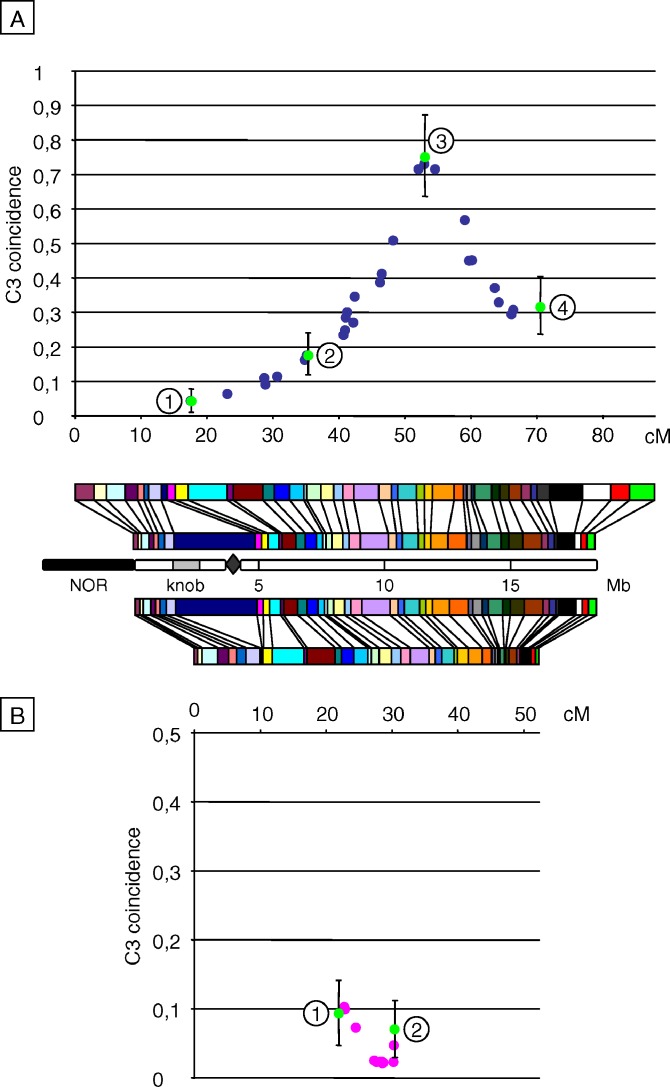
C3 Coincidence Plots along the (A) Male or (B) Female Chromosome 4 All possible pairs of adjacent intervals (each being 17.5 cM ± 5%) were used for calculation of coefficient of coincidence, defined as the frequency of double-COs divided by the frequencies of COs in both intervals (see Materials and Methods). These C3 coincidence values were plotted against the genetic position of the junction between the two adjacent intervals. Blue and pink dots represent, respectively, M and F datasets. Numbered green dots mark the pairs of intervals used for statistical comparison of C3 coincidence values. Error bar for these dots correspond to the standard deviation of coincidence calculated according to [35]. Each plot is aligned with the corresponding genetic map, the physical map, and a schematic representation of the chromosome, which includes 5-Mb scale coordinates, centromere (diamond), heterochromatic knob (gray box), nucleolar organizer region (NOR, black box).

Hence, plotting coincidence for pairs of adjacent intervals (three-point coincidence: C3; [27]) all along a chromosome gives access to local variations of interference level, provided that the genetic size of intervals remains constant. We thus performed all coincidence analyses on every possible pairs of 17.5 cM (± 5%) adjacent intervals (30 and 21 pairs fit these requirements in M and F datasets, respectively). This means that we “moved” a 2 × 17.5-cM window along the genetic map. The interference level measured by C3 was clearly variable across Chromosome 4 for both maps. In male meiosis, starting from the short-arm end, interference strength was high until ∼30 cM (C3 < 0.1), then it decreased from ∼30 cM to ∼45 cM (C3 ∼0.3), to reach a minimum at ∼52 cM (C3 ∼0.75), and finally increased again from ∼65 cM to the end of the map (C3 ∼0.3; Figure 6A). Most of these variations in C3 were found to be significant (see Figure 6, Table 1, and Materials and Methods). We can thus conclude that local interference level varied significantly along Chromosome 4 in male meiosis. In the F plot, all observed C3 values are very low (≤0.1). We could not observe any significant variation in coincidence among the few points of the plot (Figure 6 and Table 1). Given the very small number of double-COs, it seems likely that many more plants would be needed to detect any possible coincidence variation.

**Table 1:**
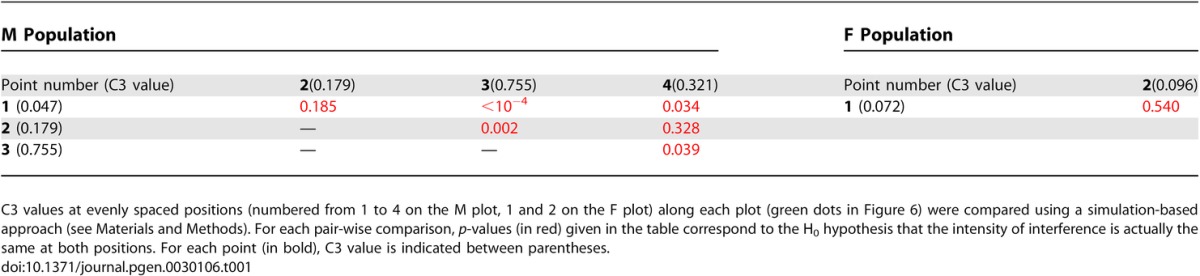
Comparison between C3 Values Obtained at Different Positions along Chromosome 4 in M and F Meiosis

### Four-Point Coefficient of Coincidence Increases with Genetic Distance

**Figure 7:**
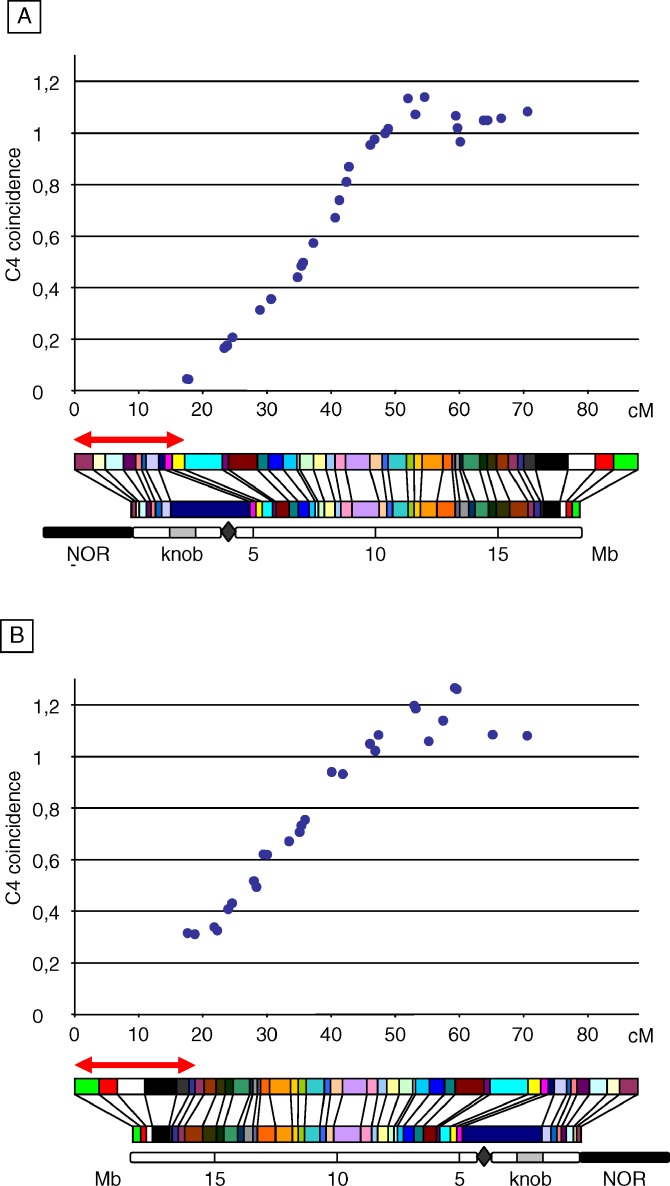
C4 Coincidence Plots along the Male Chromosome 4 All possible combinations between a fixed and a nonoverlapping interval (each being 17.5 cM ± 5%) were used for calculation of coefficient of coincidence, defined as the frequency of double-COs divided by the frequencies of COs in both intervals (see Materials and Methods). These C4 coincidence values were plotted against the genetic distance between the centers of the intervals. The fixed interval is located at the end of either (A) the short arm or (B) the long arm. Each plot is aligned with the corresponding genetic map, the physical map, and a schematic representation of the chromosome, which includes 5-Mb scale coordinates, centromere (diamond), heterochromatic knob (gray box), nucleolar organizer region (NOR, black box). For both plots, abscissa values indicate the genetic distance between the centers of the fixed and the “moving” intervals. The red two-headed arrow indicates the fixed interval position.

**Figure 8:**
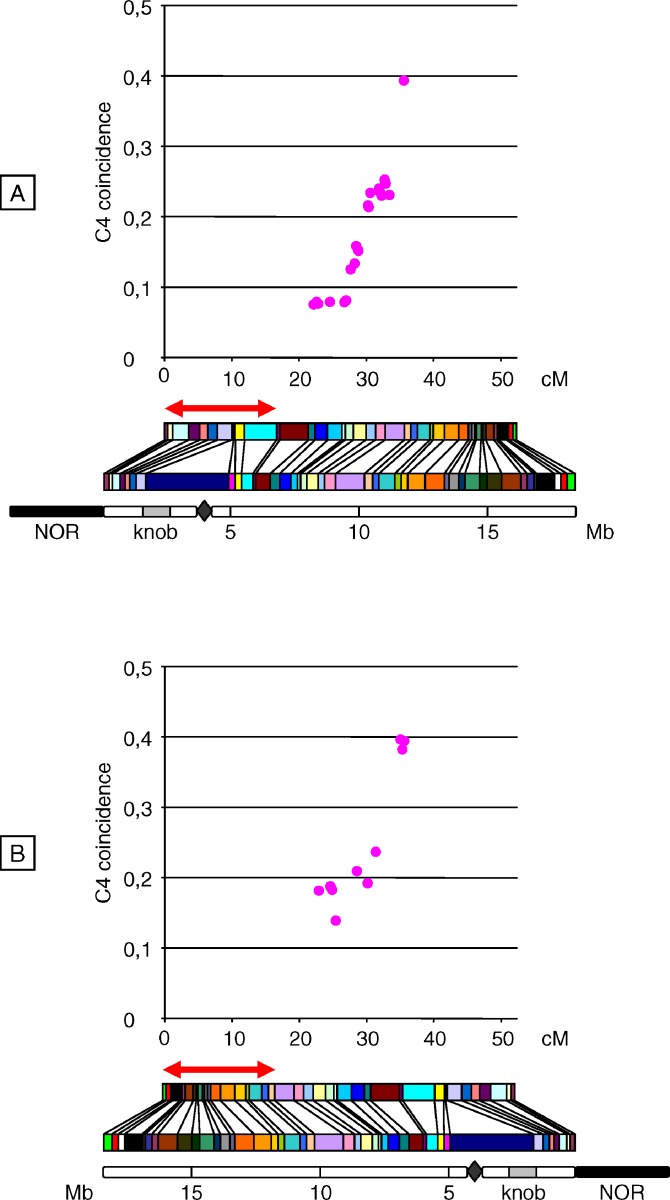
C4 Coincidence Plots along the Female Chromosome 4 All possible combinations between a fixed and a nonoverlapping interval (each being 17.5 cM ± 5%) were used for calculation of coefficient of coincidence, defined as the frequency of double-COs divided by the frequencies of COs in both intervals (see Materials and Methods). These C4 coincidence values were plotted against the genetic distance between the centers of the intervals. The fixed interval is located at the end of either (A) the short arm or (B) the long arm. Each plot is aligned with the corresponding genetic map, the physical map, and a schematic representation of the chromosome, which includes 5-Mb scale coordinates, centromere (diamond), heterochromatic knob (gray box), nucleolar organizer region (NOR, black box). For both plots, abscissa values indicate the genetic distance between the centers of the fixed and the “moving” interval. The red two-headed arrow indicates the fixed interval position.

Another way of analyzing interference is to plot coincidence between one fixed interval and a series of increasingly distant intervals (four-point coincidence: C4; [27]). This means that we fixed a 17.5 cM (± 5%) window and moved a second 17.5 cM (± 5%) window all along the chromosome. This method provides a global description of interference depending on genetic distance (see Materials and Methods). For each M and F population, two different C4 plots were made, using either the terminal interval of the short arm (Figures 7A and 8A) or the telomeric interval of the long arm as the fixed interval (Figures 7B and 8B). For the M population, regardless of whether the fixed interval was located at the end of the long or short arm, plots had globally the same shape. This kind of shape has been consistently observed in various species (for review see [41]), showing that interference decreases with genetic distance. Nevertheless, C4 values from the short-arm end were systematically lower (from 0.05 to 1.11) than those from the long-arm end (from 0.3 to 1.23). This confirms that the strength of interference is weaker at the distal region of the long arm than on the short arm. In both plots, coincidence increased up to 1 at ∼45–50 cM, peaked above 1 at ∼55–60 cM, and then decreased toward 1. The shape of the C4 plots was determined by two factors: (i) the genetic distance between intervals and (ii) fluctuations of interference strength as described above. The occurrence of a peak of coincidence above 1 means that at some genetic distances, double-COs are more frequent than expected if COs were randomly placed: this corresponds to what is called negative interference. At short distances from the terminal short-arm interval (which includes the centromere) coincidence was very low. Interestingly, this shows that the presence of the centromere did not block interference.

Regarding the F population (Figure 8), because the F map was very short (52.4 cM) and double-COs were rare, C4 plots were less informative. From both ends, coincidence increased up to ∼0.4 at ∼35 cM. Strikingly, C never reached 1, showing that in female meiosis interference acted across the whole chromosome.

### Level of Interference Covaries with Physical Size

**Figure 9:**
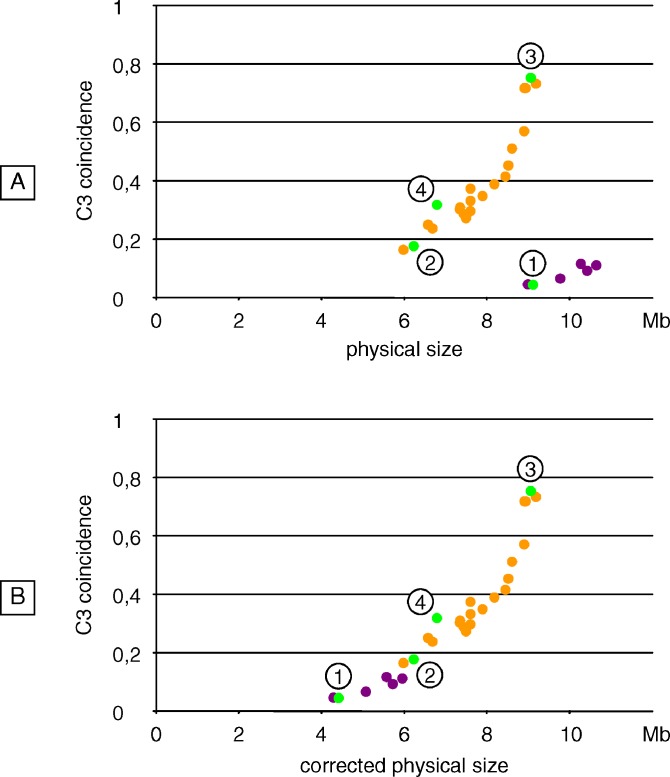
Scatter Plot between C3 Coincidence Value and Physical Size for Male Chromosome 4 Violet dots correspond to pairs of intervals encompassing the heterochromatic knob and the centromere, while orange dots correspond to the remaining pairs of intervals (all located on the long arm). Numbered green dots represent the same pairs of intervals than those represented in Figure 6A and used for the comparisons of C3 coincidence values. (A) Physical sizes were calculated from genome sequence data (Build 6 version 0), adding if necessary 1.5 Mb to take account for the centromere. (B) Cumulated centromere and knob size (4.7 Mb) was subtracted from the size of the relevant pairs of intervals.

Given that significant variations of interference level along Chromosome 4 were detected in male meiosis, we addressed the issue of possible correlations between interference level and physical distance. We thus calculated the physical size of the pairs of intervals considered for the C3 coincidence analysis described above, which have all the same genetic size (2 × 17.5 cM). The coincidence values were next plotted against these sizes (see Figure 9A). We could clearly observe two scatters of points. The smallest one contains all pairs of intervals encompassing the heterochromatic knob located on the small arm and the centromere, while the largest scatter comprises all the pairs of intervals located on the long arm only. For the large scatter, we could note a striking positive correlation between the C3 coincidence and the physical size (*r* ^2^ = 0.91 ± 0.04).

The small scatter was shifted by about 4.5 Mb relative to the large scatter. This is a direct consequence of the presence of a large CO-free region, which does not contribute to the genetic size of the considered pairs of intervals, but hugely increases their physical size. Indeed, when subtracting the cumulated size of the knob and the centromere (4.7 Mb) from the size of the pairs of intervals encompassing these chromosome parts and making a new plot (see Figure 9B), the two scatters of Figure 9A grouped into a single scatter that showed this time a chromosome-wide tight correlation between C3 coincidence and physical size (*r* ^2^ = 0.93 ± 0.04).

It means that interference level—measured by coincidence analysis on adjacent intervals of constant genetic size—decreases as the physical size increases. In other words, for a given genetic size (i.e., a given CO frequency), a greater physical size enhances the opportunity for double-COs to occur.

## Discussion

In this study, we compared male and female meiotic CO distributions along A. thaliana Chromosome 4. We depicted strong sex-related differences in CO distribution (heterochiasmy) and unequivocal variations in CO interference level along the male chromosome.

One major finding in this study was that the genetic sizes of male and female maps were strikingly different (M map 88 cM, F map 52 cM). This means that Chromosome 4 harbors an average of 1.8 CO in male meiosis, but only 1 in female meiosis. Noticeably, the size of our male map was very close to that previously reported for the Col accession [42] (87.9 versus 83.6 cM). Moreover, we described a marked difference in total SC length between sexes. Remarkably, this difference was very close to the M/F CO ratio observed for Chromosome 4. By using a high density of markers, as well as a large-sized population, we could describe the sex-specific fine scale distribution of COs for the first time in *A. thaliana.* Thus, we could detect highly significant variations of M/F CO ratio along the chromosome. We observed large differences (up to 18.7-fold) at both ends of the genetic map and less pronounced—though significant—differences on the median part of the chromosome (from 5 Mb to 13 Mb, see Figure 1C). In this median region both curves presented the same overall pattern of “peaks” and “valleys,” even if the local male CO rate was higher than the female CO rate for most intervals (30/37). In summary, we detected heterochiasmy, not only at the whole chromosome level, but also at a regional scale.

Heterochiasmy in A. thaliana has been suggested by several previous studies. Indeed, chiasma counts showed that COs are more frequent in male meiosis (9.7) than in female (8.5) [32], but the result of this count, carried out on only ten meiocytes, was not highly significant and did not provide any precise information about CO location. In two other studies, a comparison of male and female recombination rates on all five A. thaliana chromosomes was performed [29,31], but in each case only a few markers were scored, covering only part of the genome. Nevertheless, these results also suggested that recombination is higher in male than female meiosis and that there are variations in the M/F CO ratio along chromosomes. At the whole genome scale, heterochiasmy is widespread (reviewed in [1]), but this observation remains largely unexplained from an evolutionary point of view. Nevertheless, SC length was shown to differ significantly between male and female meiocytes in human [5] and mouse [6], paralleling variations in MLH1 foci number. Similar results were reported in other species, such as zebrafish [43,44], Dendrocoelum lacteum [45], and others (reviewed in [46]). This statement can now be extended to higher plants, since we obtained comparable results in *A. thaliana.* Such correlated variations in SC length and CO number were also reported among chromosomes in one sex only, among individuals in the same species, and even among meiocytes in a single organism [5,17,47–50]. However, based on current knowledge it is difficult to claim that SC length determines CO number, if the reverse is true, or even if another unidentified factor determines both.

Besides global differences, there is also compelling evidence that the distribution of COs along chromosomes is contrasted in both sexes. Similarly to what we observe in *A. thaliana,* enhancement of the M/F ratio close to telomeres was reported in other *Brassicaceae* species [2,3]. In vertebrates, such a conservation in heterochiasmy patterns along chromosomes was also observed: in mouse, human, and several teleostean fishes, the M/F ratio decreases around the centromeres and tends to increase close to the telomeres [15,16,43,51].

At the present time, the molecular and cellular bases of regional heterochiasmy remain elusive. And, generally speaking, the mechanisms ruling CO distribution along chromosomes are also poorly characterized. Data from various model organisms show that CO distribution results from the integration of several levels of control [14]: (i) the density of meiotic DSBs initiating recombination between homologous chromosomes varies along chromosomes; (ii) the propensity of a DSB to be repaired as a CO or a NCO probably varies; (iii) interference shapes the final CO distribution; (iv) only a part (variable among species) of COs are sensitive to interference (type I CO), the remaining are insensitive (type II CO). Each of these layers of control could act on observed heterochiasmy patterns along chromosomes. The DSB distribution, CO/NCO ratio, interference strength/interference strength variations, and the proportion of type II COs could vary between male and female meiosis, even if no experimental data presently support these hypotheses.

Other factors were also suggested to effect differences between CO distributions in male and female meiosis. Several studies suggested that in human, parental imprinting in a few regions could explain at least part of local heterochiasmy [52,53]. Additionally, it was proposed that synapsis initiation sites colocalize with COs [54]. For example, in human, synapsis initiation occurs in sub-telomeric regions in male [55] whereas it is rather interstitial in female (reviewed in [56]), which seems compatible with the observed pattern of heterochiasmy. Due to the availability of whole genome sequences, correlations between CO rates along chromosomes and various genomic features could be examined [15,16,19–21], but all the resulting correlations were weak. This could be explained by the fact that only sex-averaged recombination rates were used in these studies. A priori, there is no evidence that genomic features correlated to CO rates have the same weight in male and female meiosis. Thus, when possible, correlation analyses should be done separately on data from both sexes. Presumably, this could disclose previously unidentified relationships or reinforce existing ones and reveal differences in correlations between sexes.

Altogether, it is likely that multiple constraints act synergistically to shape CO distribution along male and female chromosomes in meiosis, some of which remain to be elucidated.

In this paper, we have presented the first detailed study on the effect of interference on CO distribution along a whole chromosome in male and female meiosis of *A. thaliana.* Both CO number per chromosome and inter-CO distances clearly show that COs are not independent of each other. Interestingly, we unequivocally show that the centromere is not a barrier to interference, in accordance with previous reports [18,57–59]. The coincidence plots also clearly show the existence of negative interference at some genetic distance (55–60 cM), which corresponds to a greater chance of another CO than expected from random. This phenomenon has been repeatedly observed from various genetic datasets (see, for instance, [58]) and is also predicted by various models of CO interference [27,28]. Furthermore, we provide unambiguous evidence that interference strength varies significantly along A. thaliana Chromosome 4 in male meiosis.

It has recently been shown that in most eukaryotes, a part of meiotic COs arising from a distinct pathway are not sensitive to interference [60]. Such COs account for about 15% of the total in A. thaliana [37,61]. Thus, the observed variation of interference level, measured on the whole population of COs, can be explained by two nonexclusive hypotheses: (i) the interference level between interfering COs is actually variable, or (ii) this interference level is constant, but the relative proportions of the two kinds of COs are variable along chromosome, so that locally, a high density of noninterfering COs leads to a decrease of the interference level that is measured on the whole population of COs.

In female meiosis, observed variations are not significant because double-COs are rare, hence sampling variance is high, causing an increase in *p*-values from statistical testing.

Such variations in interference strength along chromosomes were previously suggested from analysis of human pedigree data [62]. Moreover, the level of interference was reported to vary among chromosomes in humans in several studies [17,62–64]. Sex-linked variations of interference have also been reported along human Chromosome 21 [64]. Even fluctuations of interference level among human individuals have been described [17,26].

The molecular bases of these variations are currently poorly documented. Our results provide clear evidence that across a chromosome segment displaying a given CO frequency, a greater physical size enhances the opportunity for double-COs to occur. In other words, interference level between COs separated by a fixed genetic distance is a function of physical distance. Interestingly, cytogenetic data collected in humans demonstrate a negative correlation among chromosomes between SC length and the global (chromosomal) level of interference [17]. At the present time, the molecular bases of these variations are totally unknown.

The mechanisms of interference itself are still elusive. Several models have been proposed, but no experimental data directly support them. One of the most widely used is the “counting” model. Its basic postulate is that a fixed number of NCOs occurs between any two adjacent COs. As a consequence, interference strength is supposed to be constant at the chromosome scale [27,65]. Our data and those cited above strongly argue against such constancy and also call into question the concept of an unchanging “count” itself. Moreover, a recent study in yeast showed that CO number is maintained at the expense of NCOs when the DSB number is reduced, without affecting interference [66]. Other models propose that an interference signal results either from the progressive polymerization of a hypothetical structure along the chromosome [67], or from a mechanical stress imposed on the chromosome axis [28]. Our data are compatible with these two models, in which the interference level is not explicitly intended to be constant and an interference signal propagates along the chromosomes. However, as data in the field of meiotic recombination continue to accumulate exponentially, it is likely that new CO interference models supported by experimental evidences will emerge in the near future.

Our study provides the first detailed analysis of heterochiasmy and CO interference at the whole chromosome scale in a plant species. It provides the basis for future investigations on the determinism of CO distribution at the whole genome scale in A. thaliana and other species.

## Supporting Information

Table S1 List of SNP Markers Used for Genotyping Including Physical Position, 20-bp Left Flanking Sequence, and Alleles (60 KB DOC)

Table S2 Physical and Genetic Size of Intervals in Male and Female Populations (64 KB DOC)

## Abbreviations

CO: crossover
DSB: double-strand break
F: female
M: male
NCO: noncrossover
SC: synaptonemal complex
