# Supplementary material for: Sex-Specific Crossover Distributions and Variations in Interference Level along Arabidopsis thaliana Chromosome 4
Source: PLoS Genet. 2007 Jun 29;3(6):e106. doi: 10.1371/journal.pgen.0030106 (PMC1904369; doi:10.1371/journal.pgen.0030106)
Supplement: Table S1 — (60 KB DOC) [file pgen.0030106.st001.doc]

| SNP # | Physical position (pb) * | 20 bp left flanking sequence of the SNP | Col/Ler alleles | |
| --- | --- | --- | --- | --- |
| 131 | 11548 | GGGGAAGGTGTTAAAATCTC | T/A | |
| 130n | 153441 | CCACGAGCTGCGAAATACTC | C/G | |
| 128ac | 296778 | CTTAAATTTGACTTGTAACA | T/A | |
| 128 | 610274 | CTAAACTTTTATTATTGTTG | T/C | |
| 127 | 802770 | GCCTAACAGTTTCCATTTCC | C/G | |
| 126ter | 976575 | ATTAACCAACCAACGAATAG | G/A | |
| 125 | 1201323 | TAAGAACCTGAGGAATGTCT | A/T | |
| 123 | 1609256 | AACGTATATTGAGTATGGAC | A/G | |
| 103 | 4828159 | CAATGAAATTCACACTATAT | G/A | |
| 102 | 5078307 | AAGAAGACGAGGAGCAGCTT | A/G | |
| 101 | 5296115 | GCACTTCAACATCCTCATCT | G/A | |
| 99 | 5746975 | CTCGATACGGTAACGTTTCG | A/G | |
| 98 | 5850273 | CACTACACTACTTTTGGTAG | T/C | |
| 94 | 6397741 | CATCTCATCACAATAGATTC | T/C | |
| 91 | 6781817 | ATCATGAGTTGTCAGAGAGC | T/A | |
| 88 | 7234347 | GACAGACAGGTGATGCCGTT | G/A | |
| 86 | 7476193 | GAATTAAGCAGTTTCCTTTC | A/T | |
| 85 | 7600554 | GCCCAAACAAGCCCATGATA | A/G | |
| 83 | 7872867 | TTAAGCTGCTTGTCGTCAGG | G/T | |
| 80 | 8277051 | ATAGCTTGCGGTGTAAGGCA | T/C | |
| 78 | 8548281 | AATAGGATTGAAACAAAGGA | T/A | |
| 75 | 8930014 | GACATGGAAATTCCTAAACC | G/A | |
| 66 | 10104420 | TTATTATTACTACCTTAACA | C/T | |
| 64 | 10354923 | ATTGAATTTCCATGGAAGAA | G/T | |
| 62 | 10608594 | AACCTTGGACAGATTGCTCA | A/G | |
| 57 | 11255234 | GGCACGACTACTTCCACCAA | T/C | |
| 55 | 11506538 | CTGTCTATAAAAAAAAATTA | A/G | |
| 53 | 11766620 | AAGTTTGAGCAAGGCGTAAT | T/A | |
| 48 | 12407384 | TGGCGAATTTATCCTTCTAG | T/A | |
| 41 | 13170735 | TCTGCAGGTTCTTCAGGCTG | G/C | |
| 40 | 13328233 | TGGAGTGGGTATAAAGGGAG | T/C | |
| 37 | 13719691 | GACACTGTGGAAGAAAATTC | G/A | |
| 35 | 13959118 | TAGAGACCATGATTAGTAGA | G/A | |
| 31 | 14552577 | ATGAATTCCATGAAATGCAA | T/A | |
| 29 | 14819375 | CGGACATAATAGACAAGCTA | A/C | |
| 25 | 15367292 | ATATGTCTGTTGAGCATTTG | T/C | |
| 19 | 16138062 | GGGTAGAGAAAAGGAGAAGT | G/C | |
| 17 | 16390347 | TATCAACTGTTCTCACGATT | A/G | |
| 15 | 16643909 | TTCGCTTCCATACTTCTCAT | A/G | |
| 14 | 16757095 | ATTTCGTGTTTAAGAGATGC | C/A | |
| 9 | 17429315 | TTAACATGGAACTTGCGCCA | G/A | |
| 7 | 17683383 | TTGGCTTGGAGAATCCAAAT | A/G | |
| 5 | 17939118 | TAGAACTGTATATCCTATGG | C/T | |
| 2 | 18272492 | TGCAGGAAAGTTGGAGATCA | A/C | |
| * according to genome Build 6 version 0 | | |  |  |

Supplemental table 1
