# Supplementary material for: Sex-Specific Crossover Distributions and Variations in Interference Level along Arabidopsis thaliana Chromosome 4
Source: PLoS Genet. 2007 Jun 29;3(6):e106. doi: 10.1371/journal.pgen.0030106 (PMC1904369; doi:10.1371/journal.pgen.0030106)
Supplement: Table S2 — (64 KB DOC) [file pgen.0030106.st002.doc]

| Interval # | Physical size (pb) | Male genetic size (cM) | Male CO rate (cM/Mb) | Female genetic size (cM) | Female CO rate (cM/Mb) |
| --- | --- | --- | --- | --- | --- |
| 130n-131 | 141,893 | 2.889 | 20.362 | 0.613 | 4.32 |
| 128ac-130n | 143,337 | 1.973 | 13.766 | 0.613 | 4.276 |
| 128-128ac | 313,496 | 2.677 | 8.542 | 2.375 | 7.577 |
| 127-128 | 192,496 | 1.973 | 10.25 | 1.609 | 8.359 |
| 126ter-127 | 173,805 | 0.916 | 5.271 | 1.149 | 6.613 |
| 125-126ter | 224,748 | 0.704 | 3.135 | 1.609 | 7.159 |
| 123-125 | 407,933 | 1.973 | 4.837 | 2.068 | 5.071 |
| 103-123 | 3,218,903 | 0.916 | 0.284 | 0.306 | 0.095 |
| 102-103 | 250,148 | 1.268 | 5.07 | 0.229 | 0.918 |
| 101-102 | 217,808 | 1.902 | 8.735 | 1.379 | 6.332 |
| 99-101 | 450,86 | 5.778 | 12.817 | 4.75 | 10.537 |
| 98-99 | 103,298 | 0.916 | 8.868 | 0.536 | 5.192 |
| 94-98 | 547,468 | 4.721 | 8.624 | 4.214 | 7.698 |
| 91-94 | 384,076 | 1.62 | 4.22 | 0.842 | 2.194 |
| 88-91 | 452,53 | 2.325 | 5.139 | 1.992 | 4.402 |
| 86-88 | 241,846 | 2.114 | 8.741 | 2.145 | 8.871 |
| 85-86 | 124,361 | 0.493 | 3.966 | 0.459 | 3.697 |
| 83-85 | 272,313 | 2.043 | 7.504 | 1.149 | 4.22 |
| 80-83 | 404,184 | 1.902 | 4.707 | 1.992 | 4.929 |
| 78-80 | 271,23 | 1.479 | 5.456 | 1.455 | 5.367 |
| 75-78 | 381,733 | 1.62 | 4.246 | 1.226 | 3.211 |
| 66-75 | 1,174,406 | 3.805 | 3.24 | 2.911 | 2.479 |
| 64-66 | 250,503 | 1.902 | 7.595 | 0.996 | 3.976 |
| 62-64 | 253,671 | 0.986 | 3.889 | 0.919 | 3.624 |
| 57-62 | 646,64 | 2.818 | 4.359 | 1.992 | 3.081 |
| 55-57 | 251,304 | 1.198 | 4.767 | 0.536 | 2.134 |
| 53-55 | 260,082 | 1.198 | 4.606 | 1.455 | 5.597 |
| 48-53 | 640,764 | 3.453 | 5.389 | 2.222 | 3.468 |
| 41-48 | 763,351 | 1.268 | 1.661 | 1.379 | 1.806 |
| 40-41 | 157,498 | 0.563 | 3.579 | 0.459 | 2.919 |
| 37-40 | 391,458 | 0.775 | 1.98 | 0.536 | 1.37 |
| 35-37 | 239,427 | 0.493 | 2.06 | 0.229 | 0.96 |
| 31-35 | 593,459 | 2.466 | 4.156 | 0.842 | 1.42 |
| 29-31 | 266,798 | 1.198 | 4.49 | 0.229 | 0.861 |
| 25-29 | 547,917 | 1.479 | 2.7 | 0.459 | 0.839 |
| 19-25 | 770,77 | 1.832 | 2.377 | 1.149 | 1.491 |
| 17-19 | 252,285 | 1.409 | 5.586 | 0.383 | 1.518 |
| 15-17 | 253,562 | 1.127 | 4.446 | 0.153 | 0.604 |
| 14-15 | 113,186 | 1.691 | 14.942 | 0.229 | 2.031 |
| 9-14 | 672,22 | 5.073 | 7.548 | 1.149 | 1.709 |
| 7-9 | 254,068 | 4.298 | 16.919 | 0.229 | 0.904 |
| 5-7 | 255,735 | 2.818 | 11.022 | 0.689 | 2.696 |
| 2-5 | 333,374 | 3.805 | 11.415 | 0.459 | 1.379 |
| Total | 18,260,944 | 87.857 |  | 52.314 |  |

Supplemental table 2

2r
